# Fruit and vegetable consumption and all-cause mortality: evidence from a large Australian cohort study

**DOI:** 10.1186/s12966-016-0334-5

**Published:** 2016-01-25

**Authors:** Binh Nguyen, Adrian Bauman, Joanne Gale, Emily Banks, Leonard Kritharides, Ding Ding

**Affiliations:** Prevention Research Collaboration, Sydney School of Public Health, The University of Sydney, Camperdown, NSW Australia; National Centre for Epidemiology and Population Health, Research School of Population Health, Australia National University, Canberra, ACT Australia; Concord Clinical School, ANZAC Research Institute, the University of Sydney, Concord, NSW Australia

**Keywords:** Fruit, Vegetables, Mortality, Prospective studies

## Abstract

**Background:**

There is growing evidence for a relationship between fruit and vegetable consumption and all-cause mortality. Few studies, however, specifically explored consuming raw versus cooked vegetables in relation to health and mortality outcomes. The purpose of this study was to examine the relation of all-cause mortality with: a) fruit and vegetable consumption, either combined or separately; b) the consumption of raw versus cooked vegetables in a large cohort of Australian middle-aged and older adults.

**Methods:**

The sample included 150,969 adults aged 45 years and over from the 45 and Up Study, a prospective cohort study conducted in New South Wales, Australia. Self-reported baseline questionnaire data (2006–09) were linked to mortality data up to June 2014. Fruit and vegetable consumption was assessed by validated short questions. Crude and adjusted hazard ratios were calculated using Cox proportional hazard models. Covariates included socio-demographic characteristics, health-related and dietary variables.

**Results:**

During a mean follow-up of 6.2 years, 6038 (4 %) participants died from all causes. In the fully adjusted models, increasing consumption of fruit and vegetables combined was associated with reductions in all-cause mortality, with the highest risk reduction seen up to 7 serves/day or more of fruit and vegetables (*P* for trend = 0.002, hazard ratio for highest versus lowest consumption quartile: 0.90; 95 % confidence interval: 0.84, 0.97). Separate consumption of fruit and vegetables, as well as consumption of raw or cooked vegetables, were associated with a reduced risk of all-cause mortality in the crude and minimally adjusted models (all *P* for trend <0.05). With the exception of raw vegetables, these associations remained significant in the fully adjusted models (all *P* for trend <0.05). Age and sex were significant effect modifiers of the association between fruit and vegetable consumption and all-cause mortality.

**Conclusions:**

Fruit and vegetable consumption were inversely related to all-cause mortality in this large Australian cohort. Further studies examining the effects of raw versus cooked vegetables are needed.

## Background

High consumption of fruit and vegetables as part of a healthy diet is advocated for the prevention of chronic diseases, such as coronary heart disease, stroke, and some cancers [1]. Although dietary recommendations vary between countries, most are in line with the World Health Organization’s recommendation to consume a daily minimum of 400 g of fruit and vegetables, or the equivalent of five portions of fruit and vegetables per day [1–3]. Recently, a comparative risk assessment of global burden of disease identified diets low in fruit to be among the five leading risk factors worldwide [4]. Previous meta-analyses have provided evidence for the protective effects of fruit and vegetables against risk of coronary heart disease [5, 6] and stroke [7]. A recent meta-analysis has shown that the consumption of fruit and vegetables, either separately or combined, was inversely associated with a lower risk of all-cause and cardiovascular mortality [8]. The relationship with cancer mortality is less clear [8, 9], and may be specific to the kinds of fruit and vegetable consumed and types of cancers [10]. With cardiovascular disease and cancer being the primary causes of death in developed countries [11], further investigation of the protective effects of fruit and vegetables can contribute to the evidence base for public health recommendations.

To date, there has been limited research on fruit and vegetable consumption and mortality risk in Australia. Previous prospective cohort studies have been conducted in the United States, Europe and Asia [5–8]. In addition, few cohort studies have investigated the consumption of raw versus cooked vegetables in relation to mortality risk or disease incidence [9, 12]. Cooking can modify the nutritional properties of vegetables thereby influencing their potential effects on health [13]. In the large European Prospective Investigation into Cancer and Nutrition cohort, stronger inverse associations were observed for raw vegetables than for cooked vegetables with all-cause, cardiovascular and cancer mortality [9]. However, both raw and processed fruit and vegetables were not significantly related to cardiovascular disease incidence in a Dutch population-based cohort [12]. A review by Link and Potter [13] including both case–control and cohort studies showed that the consumption of raw vegetables was more strongly related to specific types of cancer than that of cooked vegetables. Although results from these observational studies tend to suggest that the associations with raw vegetables may be stronger than with cooked vegetables [9, 13], this has not been firmly established.

The aims of this paper are to examine the relation of all cause-mortality with: a) individual and combined fruit and vegetable consumption; b) the consumption of raw versus cooked vegetables, in a large Australian cohort aged 45 years and over. Findings from the current study could inform public health recommendations on fruit and vegetable consumption.

## Methods

### Study population

The Sax’s Institute’s 45 and Up Study is the largest prospective cohort study into healthy ageing in the Southern Hemisphere [14]. The cohort comprises 267,153 men and women aged 45 years and over residing in the state of New South Wales (NSW), Australia at baseline, and represents around 10 % of the NSW population aged 45 years and over. From January 2006 to December 2008, potential participants were randomly sampled from the database of Medicare Australia, the national health insurance provider and were sent an invitation to take part. The database includes Australian citizens, permanent residents, and some temporary residents and refugees. Interested participants joined the study by completing a mailed questionnaire and a consent form for follow-up which included linkage of questionnaire data to population health databases. The study methods have been described in detail elsewhere [14] and the baseline questionnaire is available at http://www.saxinstitute.org.au/our-work/45-up-study/questionnaires/. The 45 and Up Study received ethics approval from the University of NSW Human Research Ethics Committee. Approval to use data from the 45 and Up Study for this paper was obtained from the NSW Population and Health Services Ethics Committee. Participants who had reported on the baseline questionnaire that they had a history of cancer (defined as a self-reported history of cancer other than non-melanoma skin cancer: *n* = 22,900) and/or cardiovascular disease (defined as a self-reported history of heart disease, stroke or blood clot: *n* = 46,120) were excluded from the analysis. Of the remaining 203,590 participants who did not report a history of cancer and/or cardiovascular disease, 52,621 had missing data for the covariates of interest to this study and were further excluded from the analysis. The final analytic sample included 150,969 participants (83,329 women and 67,640 men).

### Measurement

#### Exposure

Self-reported baseline questionnaire data include information on socio-demographic and lifestyle factors, height and body weight, medical and surgical history, and physical functioning. Participants were asked a few dietary questions based on short validated dietary questions commonly used in health monitoring and surveillance [15]. Usual fruit consumption was assessed by asking participants: “About how many serves of fruit do you usually have each day?” with one serve of fruit corresponding to one medium piece or two small pieces of fresh fruit, or one cup of diced or canned fruit pieces. Vegetable consumption was determined from the following question: “About how many serves of vegetables do you usually eat each day?” Participants were asked to report consumption of raw and cooked vegetables separately. One serve of vegetables was defined as half a cup of cooked vegetables (including potatoes) or one cup of raw vegetables (e.g. salad). For each of these two questions, participants also had the option to answer that they did not eat fruit or vegetables, which were subsequently coded as zero serve.

#### Outcome

All-cause mortality was ascertained from the NSW Registry of Births, Deaths, and Marriages from February 1, 2006 to June 15, 2014. The mortality data were linked to the 45 and Up Study baseline data by the Centre for Health Record Linkage (CHeReL, NSW, Australia) using probabilistic record linkage methods and a commercially available software (Choice-Maker, ChoiceMaker Technologies Inc.).

#### Covariates

The following baseline self-reported variables were included as covariates: age, sex, highest educational qualification (none, school certificate, higher school certificate, trade/certificate/diploma, university degree or higher), marital status (single, widowed, divorced/separated, or married/de facto), residential remoteness (major city, regional area, or remote area), socio-economic status (quintiles based on Socio-Economic Indexes For Area - Index of Relative Socio-Economic Disadvantage [16]), smoking status (never, past, or current), hours of sleep, physical activity (assessed using validated questions from the Active Australia Survey [17]; categorised as <150, 150–300, ≥300 min per week), multi-vitamin intake (for most of the last four weeks), processed meat intake (times per week), general health (self-rated as excellent, very good, good, fair or poor), previous physician diagnosis of diabetes (yes or no) and body mass index (derived from self-reported height and weight; categorised as underweight [<18.5 kg/m^2^], normal weight [18.5–< 25.0 kg/m^2^], overweight [25.0–< 30.0 kg/m^2^], or obese [≥30.0 kg/m^2^]).

### Statistical analysis

A complete case analysis was conducted on 150,969 participants. Based on their frequency distribution, fruit and vegetable consumption were categorised as quartiles (Q). Descriptive statistics for baseline characteristics were computed for the overall sample and according to fruit and vegetable consumption, both as separate and combined categories. Crude and adjusted hazard ratios (HRs) with 95 % confidence intervals (CIs) were estimated for all categories of fruit and vegetable consumption (combined fruit and vegetables, total fruit, total vegetables, cooked vegetables, raw vegetables) by using Cox proportional hazards models, with the lowest intake category used as a reference category. To test the statistical significance for trends (measured by probability [*P*] for trends) in the associations across increasing quartiles of fruit and vegetable intake, quartiles of intake were replaced with a continuous variable calculated from the respective midpoints of the quartiles in the Cox proportional hazard models. Three models were tested for each exposure variable: Model 1 was an unadjusted model; Model 2 was minimally adjusted for age and sex; Model 3 was adjusted for age, sex, education level, marital status, location of residence, socio-economic status, smoking status, physical activity categories, multi-vitamin use, processed meat consumption, diabetes and body mass index categories. Analyses of vegetable consumption were adjusted for fruit consumption (and vice versa).

As suggested by previous studies, variables including age, sex, education level, smoking and body mass index could potentially moderate the association between fruit and vegetable consumption and all-cause mortality [9, 12]. Furthermore, lifestyle factors, such as fruit and vegetable consumption, may have differential effects on those with different health status. Therefore, in Model 3, we tested for potential effect modification by age group (45–59 years; 60–74 years; ≥75 years), sex, education level, smoking status, body mass index categories, and self-rated health. Any significant (*P* < 0.05) interactions were further explored in stratified analyses. Finally, due to the relatively short follow-up, an additional sensitivity analysis was conducted by repeating the analysis on those with at least two years of follow-up to test for occult disease at baseline. Data were analysed using SAS version 9.3 (SAS Institute Inc.) and the significance level was set at 0.05.

## Results

### Participant characteristics

Among 150,969 participants followed up for an average of 6.2 (standard deviation [SD]: 1.0) years and a total of 933,538 person-years, 6,038 (4 %) died. Overall, the mean age (SD) of participants at baseline was 60.0 (10.1) years, more than half (55.2 %) of participants were women, more than a quarter (27 %) completed college or university, more than three quarters (77.7 %) were in a married/de facto relationship, and 54.7 % lived in regional/remote areas. The mean intakes (SD) for fruit, vegetables, and both fruit and vegetables, were respectively: 1.9 (1.4), 3.9 (2.6), and 5.8 (3.3) servings/day. More than half (60.5 %) of the sample (48.6 % of men; 70.2 % of women) met the World Health Organization’s recommendations of consuming a combination of five serves of fruit and vegetables per day [1]. Baseline characteristics of study participants by categories of fruit and vegetable consumption are presented in Table 1. Compared with participants with lower intakes of fruit and vegetables, those who consumed higher amounts were more likely to be younger, women, in a married/de facto relationship, and living in remote/rural areas. Such participants were also more likely to sleep between 7 to 9 h per day, exercise more than 300 min/week, be non-smokers, non-obese and to perceive themselves in very good or excellent health.

**Table 1:** Baseline characteristics of 150,969 participants from the 45 and Up Study by frequency of fruit and vegetable intakes^a^

		Quartiles of fruit intake^b^				Quartiles of vegetable intake^b^				Quartiles of combined fruit and vegetable intake^b^			
Variable	Overall	Q1	Q2	Q3	Q4	Q1	Q2	Q3	Q4	Q1	Q2	Q3	Q4
Number of subjects	150,969	12,960	50,766	50,111	37,132	54,562	24,460	40,979	30,968	37,467	43,004	34,779	35,719
Mean servings per day (SD)		0.001 (0.02)	1.01 (0.05)	2.00 (0.00)	3.73 (1.46)	1.65 (0.57)	2.99 (0.08)	4.39 (0.49)	7.83 (2.61)	2.44 (0.80)	4.49 (0.50)	6.44 (0.50)	10.27 (3.12)
Age group (%)													
45 to 59 years	56.5	64.5	58.3	55.6	52.4	59.1	59.4	55.2	51.3	59.9	59.4	55.2	50.7
60 to 74 years	33.5	28.5	32.0	34.3	36.3	30.7	31.1	35.2	38.0	30.1	31.2	35.1	38.2
≥75 years	10.0	7.1	9.7	10.1	11.3	10.3	9.4	9.6	10.7	10.0	9.4	9.7	11.1
Women (%)	55.2	41.2	48.0	61.4	61.6	40.5	55.3	65.7	67.1	36.6	54.1	64.8	66.7
College or higher education (%)	27.0	18.0	26.0	28.5	29.5	26.1	31.9	29.0	22.1	24.1	29.8	29.1	24.7
Married/de facto (%)	77.7	73.0	78.8	78.6	76.8	74.7	78.6	80.3	78.9	74.3	78.4	79.9	78.4
Location of residence													
Major city (%)	45.3	42.2	44.5	45.5	47.0	47.9	47.6	44.3	40.1	46.9	46.9	44.9	41.9
Rural/remote (%)	54.7	57.8	55.5	54.5	53.0	52.1	52.4	55.7	59.9	53.1	53.1	55.1	58.1
Socio-economic status (SEIFA-IRSD) (%)													
Quintile 1 (most disadvantaged)	19.1	22.6	20.0	18.1	17.9	19.1	17.5	18.3	21.3	20.0	18.2	18.1	20.2
Quintile 2	19.1	20.7	19.0	19.1	18.8	18.8	18.4	19.1	20.2	19.0	18.7	18.9	19.8
Quintile 3	21.0	21.4	21.1	21.0	20.5	20.6	20.1	21.1	22.2	20.9	20.2	21.2	21.7
Quintile 4	20.1	19.4	19.6	20.5	20.6	20.0	20.6	20.6	19.3	19.8	20.4	20.5	19.7
Quintile 5 (least disadvantaged)	20.7	15.9	20.2	21.4	22.2	21.4	23.4	21.0	17.0	20.3	22.5	21.3	18.6
Current smoking (%)	7.5	21.2	9.2	5.0	3.8	9.9	6.6	5.8	6.2	12.7	7.0	5.3	4.9
Hours of sleep (%)													
<7 h/day	15.1	19.8	15.2	13.9	15.0	16.9	13.9	13.5	15.0	17.3	14.7	13.6	14.7
7–9 h/day	67.6	60.7	66.9	69.5	68.6	66.3	69.1	68.8	67.3	65.0	68.7	68.9	68.0
≥9 h/day	17.3	19.6	17.8	16.7	16.4	16.7	17.0	17.7	17.7	17.7	16.6	17.5	17.3
Physical activity category (%)													
10–149 min/week	19.1	29.0	21.9	16.9	14.8	22.9	18.5	16.7	16.1	25.7	19.0	16.0	15.2
150–299 min/week	16.1	17.2	17.2	15.9	14.3	17.0	17.2	15.8	13.8	17.5	17.0	15.8	13.7
≥300 min/week	64.8	53.8	61.0	67.2	70.8	60.0	64.4	67.5	70.1	56.7	64.0	68.1	71.1
Multivitamin use (%)	3.2	2.9	3.3	3.2	3.1	3.3	3.1	2.9	3.1	3.3	3.2	2.9	3.1
Self-rated health (%)													
Excellent	18.3	12.4	15.7	19.2	22.8	15.7	18.7	20.0	20.4	13.8	18.1	20.0	21.7
Very good	40.6	33.2	40.0	42.2	41.7	38.4	40.8	42.3	42.0	36.8	41.1	42.6	42.0
Good	31.4	37.0	33.7	30.2	27.7	34.0	31.4	29.5	29.1	35.9	31.6	29.3	28.3
Fair	8.5	14.7	9.3	7.4	6.9	10.4	8.1	7.2	7.6	11.7	8.2	7.3	7.0
Poor	1.2	2.7	1.3	0.9	0.9	1.5	1.0	0.9	1.0	1.8	1.1	0.8	0.9
BMI category (%)													
Underweight and normal weight (≤18.5 to <25.0 kg/m^2^)	39.0	34.9	37.7	39.1	42.1	37.3	41.1	40.3	38.7	36.5	39.3	40.3	40.0
Overweight (25.0 to <30.0 kg/m^2^)	39.4	39.3	40.5	39.2	38.0	41.5	38.8	37.9	37.9	41.6	39.8	38.0	37.8
Obese (≥30.0 kg/m^2^)	21.6	25.8	21.8	21.7	19.9	21.2	20.1	21.8	23.4	21.8	21.0	21.7	22.2
Physician diagnosed diabetes (%)	7.0	5.9	6.4	7.4	7.6	6.9	6.3	6.9	7.7	6.5	6.7	7.0	7.7

*Abbreviations*: *BMI* body mass index, *min* minutes, *IRSD* Index of Relative Socio-economic Disadvantage, *Q* quartile of intake, *SD* standard deviation, *SEIFA* Socio-Economic Indexes For Areas

^a^Data are presented as means (SD) or percentages (%)

^b^The quartiles of intake for fruit and vegetables (servings/day) were as follows: Fruit: Q1: <1.0; Q2: 1.0 to <2.0; Q3: 2.0 to <2.3; Q4: ≥2.3. Vegetables: Q1: ≤2.0; Q2: 2.0 to ≤3.0; Q3: 3.0 to ≤5.0, Q4: >5.0. Fruit and vegetables combined: Q1: <4.0; Q2: 4 to ≤5.0; Q3: >5.0 to ≤7.0; Q4: >7.0

### Fruit and vegetable consumption and all-cause mortality

Table 2 shows the HRs for all-cause mortality according to categories of fruit and vegetable intake. The combined consumption of fruit and vegetables was inversely related to all-cause mortality in all models. In the fully adjusted model, this association was substantially attenuated compared with the unadjusted model (Q4 versus Q1; HR: 0.76; 95 % CI: 0.71, 0.81; *P* for trend < 0.0001) but remained significant (Q4 versus Q1; HR: 0.90; 95 % CI: 0.84, 0.97; *P* for trend = 0.002). The highest risk reduction was observed with the highest consumption quartile (Q4: 7 serves or more of fruit and vegetables/day).

**Table 2:** Hazard ratios and 95 % confidence intervals of all-cause mortality by quartiles of intake for fruit and vegetables (*n* = 150,969)

	Quartiles^a^								
	Q1		Q2		Q3		Q4		P for trend
	HR	95 % CI	HR	95 % CI	HR	95 % CI	HR	95 % CI	
Fruit and vegetable intake^a^									
Model 1 (crude)	1.0	Reference	0.80	0.75,0.85	0.70	0.65,0.75	0.76	0.71,0.81	<0.0001
Model 2^b^ (age, sex adjusted)	1.0	Reference	0.89	0.83,0.95	0.79	0.73,0.85	0.77	0.72,0.83	<0.0001
Model 3^c^ (adjusted)	1.0	Reference	0.99	0.93,1.06	0.92	0.86,0.99	0.90	0.84,0.97	0.002
Fruit intake^a^									
Model 1 (crude)	1.0	Reference	0.91	0.83,1.00	0.78	0.72,0.86	0.78	0.71,0.85	<0.001
Model 2^b^ (age, sex adjusted)	1.0	Reference	0.75	0.69,0.83	0.66	0.60,0.72	0.62	0.56,0.68	<0.001
Model 3^c^ (adjusted)	1.0	Reference	0.91	0.83,0.99	0.86	0.78,0.94	0.84	0.76,0.93	0.001
Vegetable intake^a^									
Model 1 (crude)	1.0	Reference	0.78	0.72,0.84	0.71	0.66,0.75	0.79	0.74,0.85	<0.0001
Model 2^b^ (age, sex adjusted)	1.0	Reference	0.87	0.81,0.94	0.81	0.76,0.87	0.82	0.77,0.88	<0.0001
Model 3^c^ (adjusted)	1.0	Reference	0.95	0.88,1.02	0.92	0.86,0.99	0.93	0.87,1.00	0.017
Cooked vegetable intake^a^									
Model 1 (crude)	1.0	Reference	0.74	0.68,0.80	0.87	0.81,0.94	0.88	0.81,0.95	0.004
Model 2^b^ (age, sex adjusted)	1.0	Reference	0.86	0.80,0.93	0.89	0.83,0.97	0.80	0.74,0.86	<0.0001
Model 3^c^ (adjusted)	1.0	Reference	0.92	0.85,1.00	0.98	0.90,1.06	0.87	0.80,0.95	0.003
Raw vegetable intake^a^									
Model 1 (crude)	1.0	Reference	0.62	0.57,0.66	0.56	0.50,0.61	0.65	0.59,0.72	<0.0001
Model 2^b^ (age, sex adjusted)	1.0	Reference	0.76	0.70,0.82	0.76	0.69,0.84	0.77	0.70,0.85	0.0005
Model 3 (adjusted)	1.0	Reference	0.87	0.81,0.94	0.92	0.84,1.02	0.94	0.85,1.04	0.793

*Abbreviations*: *CI* confidence interval, *HR* hazard ratio, *Q* quartile

^a^The quartiles of intake for fruit and vegetables (servings/day) were as follows: Fruit and vegetables combined: Q1: <4.0; Q2: 4 to ≤ 5.0; Q3: 5.0 to ≤7.0; Q4: >7.0. Fruit: Q1: <1.0; Q2: 1.0 to <2.0; Q3: 2.0 to <2.3; Q4: ≥2.3. Vegetables: Q1: ≤2.0; Q2: 2.0 to ≤3.0; Q3: 3.0 to ≤5.0, Q4: >5.0. Cooked vegetables: Q1: ≤1.0; Q2: 1.0 to ≤2.0; Q3: 2.0 to ≤3.0, Q4: >3.0. Raw vegetables: Q1: <1.0; Q2: 1.0 to <1.3; Q3: 1.3 to ≤ 2.0; Q4: >2.0

^b^Model 2 was adjusted for age (continuous) and sex

^c^Model 3 was adjusted for age (categorical), sex, education level, marital status, location of residence, socio-economic status, smoking status, physical activity categories, multi-vitamin use, processed meat consumption, diabetes and body mass index categories. Any significant (*P* < 0.05) interactions (shown in Table 3) with age group, sex, education level, body mass index categories and smoking status, were included in this model. The model for fruit was adjusted for vegetable intake and vice versa

Similarly, when fruit consumption was considered separately, there was an inverse association with all-cause mortality in all models. Participants in the top quartile had a significantly lower risk of all-cause mortality than those in the bottom quartile (fully adjusted model: HR: 0.84; 95 % CI: 0.76, 0.93; *P* for trend ≤ 0.001). Consumption of total vegetables, as well as separate consumption of cooked and raw vegetables, was associated with a lower risk of all-cause mortality in the unadjusted and minimally adjusted models (all *P* for trend < 0.05). In the fully adjusted models, these associations were markedly attenuated compared with the unadjusted models, but remained statistically significant for total vegetables and cooked vegetables only (P for trend < 0.05). The association with raw vegetable consumption showed estimates (and CIs) that were consistent with those for cooked vegetables but these findings were not significant. The sensitivity analyses conducted on participants with at least two years of follow-up showed similar results that were slightly attenuated (Appendix: Table 4).

### Effect modification

Significant (*P* < 0.05) effect modifiers of the association between fruit and vegetable intake and risk of all-cause mortality included sex and age group (Table 3). Consumption of fruit and vegetables combined, and separate consumption of vegetables, were inversely related with all-cause mortality in women but not in men. Consumption of fruit was associated with lower HRs in individuals aged between 60 to 74 years compared to other age groups.

**Table 3:** Hazard ratios and 95 % confidence intervals of all-cause mortality by quartiles of intake for fruit and vegetables by effect modifiers

Effect value for significant interactions	Quartiles^a^								
	Q1		Q2		Q3		Q4		P for interaction
	HR	95 % CI	HR	95 % CI	HR	95 % CI	HR	95 % CI	
Fruit and vegetable intake^a^									
Male	1.0	Reference	1.03	(0.95,1.12)	0.97	(0.88,1.06)	1.01	(0.92,1.11)	0.002
Female	1.0	Reference	0.89	(0.79,0.99)	0.80	(0.71,0.91)	0.76	(0.67,0.85)	
Fruit intake^a^									
45 to 59 years	1.0	Reference	0.83	(0.68,1.01)	0.88	(0.72,1.08)	0.86	(0.69,1.07)	0.045
60 to 74 years	1.0	Reference	0.84	(0.73,0.98)	0.80	(0.68,0.93)	0.82	(0.69,0.96)	
≥75 years	1.0	Reference	1.13	(0.98,1.30)	1.05	(0.91,1.21)	0.98	(0.84,1.13)	
Vegetable intake^a^									
Male	1.0	Reference	0.94	(0.86,1.04)	0.94	(0.86,1.03)	1.04	(0.94,1.14)	0.012
Female	1.0	Reference	0.94	(0.77,1.14)	0.84	(0.76,0.93)	0.82	(0.73,0.92)	

*Abbreviations*: *CI* confidence interval, *HR* hazard ratio, *Q* quartile

^a^The quartiles of intake for fruit and vegetables (servings/day) were as follows: Fruit and vegetables combined: Q1: <4.0; Q2: 4 to ≤ 5.0; Q3: 5.0 to ≤7.0; Q4: >7.0. Fruit: Q1: <1.0; Q2: 1.0 to <2.0; Q3: 2.0 to <2.3; Q4: ≥2.3. Vegetables: Q1: ≤2.0; Q2: 2.0 to ≤3.0; Q3: 3.0 to ≤5.0, Q4: >5.0

## Discussion

To our knowledge, this is the first prospective cohort study in Australia and one of the largest worldwide to explore the relationship between fruit and vegetable consumption and all-cause mortality. Consumption of fruit and vegetables combined was inversely related to all-cause mortality in this large cohort of middle-aged and older Australian adults. After adjustment for age and sex, this association was attenuated by approximately 6 %. Following adjustment for socio-economic, lifestyle and health-related factors, the association was attenuated further by approximately 12 % but remained statistically significant. Individual consumption of fruit or vegetables was associated with reduced mortality from all-causes in all models. Vegetables consumed cooked or raw, were also associated with a lower risk of all-cause mortality in unadjusted and minimally adjusted models. However, after adjustment for all other covariates, cooked vegetables remained significantly related to a lower risk of all-cause mortality. While the association of raw vegetables with all-cause mortality was similar to that of cooked vegetables, it did not remain statistically significant. Sex and age group were significant effect modifiers of the relationship between fruit and vegetable consumption and all-cause mortality.

Findings from the present study are in line with those from previous prospective cohort studies which have mostly found a significant inverse relationship between fruit and vegetable intake (considered separately or combined) and all-cause mortality [9, 18–20]. A recent meta-analysis of prospective cohort studies showed that pooled hazard ratios of all-cause mortality were 0.95 (95 % confidence interval: 0.92, 0.98; *P* = 0.001) for an increment of one serving a day of fruit and vegetables, 0.94 (95 % CI: 0.90, 0.98; *P* = 0.002) for fruit, and 0.95 (95 % CI: 0.92, 0.99; *P* = 0.006) for vegetables [8]. In our study, the protective effect of consuming both fruit and vegetables was slightly smaller. Differences in findings could be due to a number of factors that vary between studies including measures of fruit and vegetable consumption, covariate adjustment, follow-up time and cohort characteristics. Previous studies generally had longer follow-up periods and more detailed dietary measures [8]. However, in their meta-analysis, Wang et al. found that study location, sex, sample size, study quality and duration of follow-up had little impact on the association between fruit and vegetable intake and all-cause mortality [8].

The meta-analysis by Wang and colleagues also showed a dose–response relationship up to a threshold of five servings/day for fruit and vegetables combined, two servings/day for fruit, and three servings/day for vegetables [8]. There was no further reduction in mortality risk beyond these thresholds. In the present study, protective effects on mortality risk were observed starting with the second quartile of consumption (4 to ≤5 servings/day of fruit and vegetables, 1 to <2 servings/day of fruit and 2 to ≤3 servings/day of vegetables). However, with the exception of vegetable consumption which appeared to reach a threshold at 3 to ≤5 servings/day beyond which there was no further risk reduction, findings from the present study differed from those of the meta-analysis. For the consumption of fruit and vegetables combined and that of fruit only, the point estimates decreased with increasing serves, with the highest risk reductions achieved with 7 serves/day or more of fruit and vegetables, and 2.3 serves/day or more of fruit. These findings are in agreement with a recent study conducted in the United Kingdom which found a strong inverse association between combined fruit and vegetable consumption and all-cause mortality in 65,226 participants aged 35 years and over, with highest benefits seen with 7 serves/day or more of fruit and vegetables, 3 to <4 serves/day of fruit and 3 serves/day or more of vegetables [19]. It seems that current Australian recommendations to consume two serves of fruit (150 g each) and five serves of vegetables (75 g each) per day are appropriate [21]. However, more efforts at promoting fruit and vegetable consumption are needed as approximately only 6–8 % of the NSW population aged 45 years and over currently meet Australian recommendations for fruit and vegetable intake [22].

To date, several observational studies have investigated the differential effects of consuming cooked versus raw vegetables on all-cause mortality. Cooking can alter the physical structure and properties of bio-active compounds (such as phytochemicals, vitamins, minerals and fibre) contained in vegetables, and thereby change their physiologic effect, in a potentially beneficial or less desirable way for health [13]. For example, the bioavailability of compounds that may act as antioxidants can either be enhanced (e.g. certain carotenoids such as carotenes in carrots and lycopene in tomatoes) or decreased (e.g. vitamin C) by heat treatment. Although results are still preliminary, several observational studies found that raw vegetable consumption was more protective against mortality than cooked vegetable consumption [9, 13]. The results of this study show similar relationships of cooked and raw vegetables to mortality. It should be noted that power was limited for raw vegetable consumption in the current study, which made it difficult to detect significant differences between quartiles. Further studies examining the effects of raw versus cooked vegetables on mortality risk are needed to explore these preliminary findings.

While there were some significant effect modifiers of the association between fruit and vegetable consumption and all-cause mortality identified in the current study, these findings should be interpreted with caution. Consumption of fruit and vegetables combined, as well as separate consumption of vegetables only, were inversely related with risk of all-cause mortality in women but not in men. To date, sex differences in these associations have not been clearly established. The meta-analysis by Wang et al. as well as several previous studies did not observe significant sex differences [8, 12, 18, 23, 24]. However, in the large European Prospective Investigation into Cancer and Nutrition cohort study, while there were no significant sex differences for the consumption of fruit and vegetables combined or the individual consumption of vegetables, the individual consumption of fruit was inversely associated with risk of all-cause mortality in women but not in men [9]. As suggested by the authors of that study, possible explanations for the discrepancy in findings include residual confounding, a finding due to chance, or a true biological difference, although a mechanism for such a difference is not apparent. Women may also report their intake of fruit and vegetables more accurately than men. In the few studies that stratified analyses by age, there was no significant effect modification by age [12, 25]. Clearly, further evidence is needed to support findings about potential effect modifiers and the underlying mechanisms remain to be elucidated.

### Strengths and limitations

The main strengths of this study include a large population-based sample, high quality record linkage and the inclusion of multiple socio-demographic, health-related and dietary covariates. The processing of vegetables (i.e., whether vegetables were cooked or raw) was also considered. The prospective nature of the study helped minimise recall bias.

This study had several limitations including the relatively short follow-up time which may have been insufficient to observe long-term effects of fruit and vegetable intake. Although the short dietary questions used in this study are appropriate for large-scale studies, it is possible that the self-reported consumption may not accurately capture true consumption. Most previous studies have used more detailed dietary methods such as food frequency questionnaires and food records, although these are also prone to measurement error [8]. One major limitation of our brief questionnaire in comparison with more detailed questionnaires such as food frequency questionnaires, is that we did not measure the specific fruits and vegetables consumed. Examining the roles of different types of fruit and vegetables could be important as some kinds of fruit and vegetables could be more beneficial than others. In addition, the dietary questions were asked only at baseline and may not reflect the long-term habitual patterns of dietary behaviour. As this is an observational study, residual confounding could also be of concern. We tried to minimise this by adjusting for multiple covariates and by repeating the analyses among those with at least two years of follow-up data. Due to the limited number of dietary questions in the 45 and Up Study questionnaire, we could not assess other food items beyond processed meat as potential confounders. Future studies could consider including more detailed dietary measures differentiating between subgroups of vegetables, whether vegetables are consumed raw or cooked, cooking method, and collecting repeated dietary measures over time to establish long-term patterns of fruit and vegetable consumption.

## Conclusions

In this large cohort of middle-aged and older Australian adults, consumption of fruit and vegetables was inversely associated with all-cause mortality during 6.2 years of follow-up. Findings from this study support recommendations to consume a high amount of fruit and vegetable consumption. The association of raw versus cooked vegetables in relation to mortality requires further investigation.

## Appendix

**Table 4:** Hazard ratios and 95 % confidence intervals of all-cause mortality by quartiles of intake for fruit and vegetables for sensitivity analyses conducted on 149,787 participants with at least two years of follow-up

	Quartiles^a^								
	Q1		Q2		Q3		Q4		P for trend
	HR	95 % CI	HR	95 % CI	HR	95 % CI	HR	95 % CI	
Fruit and vegetable intake^a^									
Model 1 (crude)	1.0	Reference	0.81	0.76, 0.88	0.74	0.68, 0.80	0.80	0.74, 0.86	<0.0001
Model 2^b^ (age, sex adjusted)	1.0	Reference	0.90	0.84, 0.97	0.83	0.76, 0.90	0.80	0.74, 0.87	<0.0001
Model 3^c^ (adjusted)	1.0	Reference	1.00	0.93, 1.08	0.96	0.88, 1.04	0.93	0.86, 0.93	0.07
Fruit intake^a^									
Model 1 (crude)	1.0	Reference	0.90	0.81, 0.99	0.79	0.71, 0.87	0.79	0.71, 0.88	<0.001
Model 2^b^ (age, sex adjusted)	1.0	Reference	0.74	0.67, 0.82	0.66	0.59, 0.73	0.62	0.56, 0.69	<0.001
Model 3^c^ (adjusted)	1.0	Reference	0.88	0.80, 0.98	0.84	0.76, 0.94	0.83	0.74, 0.93	0.003
Vegetable intake^a^									
Model 1 (crude)	1.0	Reference	0.82	0.75, 0.89	0.73	0.68, 0.79	0.84	0.78, 0.91	<0.0001
Model 2^b^ (age, sex adjusted)	1.0	Reference	0.91	0.83, 0.99	0.84	0.78, 0.90	0.87	0.80, 0.94	<0.0001
Model 3^c^ (adjusted)	1.0	Reference	0.98	0.90, 1.07	0.94	0.87, 1.02	0.98	0.90, 1.06	0.309

*Abbreviations*: *CI* confidence interval, *HR* hazard ratio, *Q* quartile

^a^The quartiles of intake for fruit and vegetables (servings/day) were as follows: Fruit and vegetables combined: Q1: <4.0; Q2: 4 to ≤ 5.0; Q3: 5.0 to ≤7.0; Q4: >7.0. Fruit: Q1: <1.0; Q2: 1.0 to <2.0; Q3: 2.0 to <2.3; Q4: ≥2.3. Vegetables: Q1: ≤2.0; Q2: 2.0 to ≤3.0; Q3: 3.0 to ≤5.0, Q4: >5.0

^b^Model 2 was adjusted for age (continuous) and sex

^c^Model 3 was adjusted for age (categorical), sex, education level, marital status, location of residence, socio-economic status, smoking status, physical activity categories, multi-vitamin use, processed meat consumption, diabetes and body mass index categories. The model for fruit was adjusted for vegetable intake and vice versa

## Abbreviations

CI: confidence interval
HR: hazard ratio
NSW: State of New South Wales
P: probability
Q: quartile
SD: standard deviation

## References

[1] Joint WHO/FAO Expert Consultation (2003). Diet, nutrition and the prevention of chronic diseases. World Health Organ Tech Rep Ser.

[2] National Health Service, United Kingdom government. 5 a day. http://www.nhs.uk/livewell/5aday/Pages/5ADAYhome.aspx. Accessed May 29, 2015.

[3] Programme National Nutrition Santé, French Government. Fruits et légumes: au moins 5 par jour. http://www.mangerbouger.fr/bien-manger/que-veut-dire-bien-manger-127/les-9-reperes/fruits-et-legumes-au-moins-5-par-jour.html. Accessed May 29, 2015.

[4] Lim SS, Vos T, Flaxman AD, Danaei G, Shibuya K, Adair-Rohani H (2012). A comparative risk assessment of burden of disease and injury attributable to 67 risk factors and risk factor clusters in 21 regions, 1990–2010: a systematic analysis for the Global Burden of Disease Study 2010. Lancet.

[5] Dauchet L, Amouyel P, Hercberg S, Dallongeville J (2006). Fruit and vegetable consumption and risk of coronary heart disease: a meta-analysis of cohort studies. J Nutr.

[6] He FJ, Nowson CA, Lucas M, MacGregor CA (2007). Increased consumption of fruit and vegetables is related to a reduced risk of coronary heart disease: meta-analysis of cohort studies. J Hum Hypertens.

[7] He FJ, Nowson CA, MacGregor GA (2006). Fruit and vegetable consumption and stroke: meta-analysis of cohort studies. Lancet.

[8] Wang X, Ouyang Y, Liu J, Zhu M, Zhao G, Bao W (2014). Fruit and vegetable consumption and mortality from all causes, cardiovascular disease, and cancer: systematic review and dose–response meta-analysis of prospective cohort studies. BMJ.

[9] Leenders M, Sluijs I, Ros MM, Boshuizen HC, Siersema PD, Ferrari P (2013). Fruit and vegetable consumption and mortality European Prospective Investigation into Cancer and Nutrition. Am J Epidemiol.

[10] World Cancer Research Fund and the American Institute for Cancer Research (2007). Food, Nutrition, Physical Activity, and the Prevention of Cancer: A Global Perspective.

[11] World Health Organization. The top 10 causes of death, 2012. www.who.int/mediacentre/factsheets/fs310/en. Accessed September 24, 2015.

[12] Oude Griep LM, Geleijnse JM, Kromhout D, Ocke MC, Verschuren WMM (2010). Raw and processed fruit and vegetable consumption and 10-Year coronary heart disease incidence in a population-based cohort study in the Netherlands. PLoS One.

[13] Link LB, Potter JD (2004). Raw versus cooked vegetables and cancer risk. Cancer Epidemiol Biomarkers Prev.

[14] Banks E, Redman S, Jorm L, Armstrong B, Bauman A, Beard J (2008). Cohort profile: the 45 and up study. Int J Epidemiol.

[15] Rustihauser IHE, Webb K, Abraham B, Allsopp R (2011). Evaluation of short dietary questions from the 1995 NNS.

[16] Australian Bureau of Statistics. Information paper: an introduction to Socio-Economic Indexes for Areas (SEIFA) 2006*.* Canberra, ACT: Australian Bureau of Statistics; 2008. (Information Paper Cat No: 2039.0.).

[17] Australian Institute of Health and Welfare (2003). The Active Australia Survey: a guide and manual for implementation, analysis and reporting.

[18] Bellavia A, Larsson SC, Bottai M, Wolk A, Orsini N (2013). Fruit and vegetable consumption and all-cause mortality: a dose–response analysis. Am J Clin Nutr.

[19] Oyebode O, Gordon-Dseagu V, Walker A, Mindell JS (2014). Fruit and vegetable consumption and all-cause, cancer and CVD mortality: analysis of Health Survey for England data. J Epidemiol Community Health.

[20] Zhang X, Shu XO, Xiang YB, Yang G, Li H, Gao J (2011). Cruciferous vegetable consumption is associated with a reduced risk of total and cardiovascular disease mortality. Am J Clin Nutr.

[21] Australian Department of Health and Ageing, Australian Government. Go for 2&5. http://www.healthyactive.gov.au/internet/healthyactive/publishing.nsf/Content/2and5. Accessed May 29, 2015.

[22] Australian Bureau of Statistics. Australian Health Survey: First results, 2011–12, Cat No: 4364.0.55.001, Table 10: Daily intake of fruit and vegetables by age and sex – Australia. http://www.abs.gov.au/AUSSTATS/abs@.nsf/DetailsPage/4364.0.55.0012011-12?OpenDocument. Accessed July 20, 2015.

[23] Genkinger JM, Platz EA, Hoffman SC, Comstock GW, Helzlsouer KJ (2004). Fruit, vegetable, and antioxidant intake and all-cause, cancer, and cardiovascular disease mortality in a community-dwelling population in Washington County, Maryland. Am J Epidemiol.

[24] Nagura J, Iso H, Watanabe Y, Maruyama K, Date C, Toyoshima H (2009). Fruit, vegetable and bean intake and mortality from cardiovascular disease among Japanese men and women: the JACC Study. Br J Nutr.

[25] Nakamura K, Nagata C, Oba S, Takatsuka N, Shimizu H (2008). Fruit and vegetable intake and mortality from cardiovascular disease are inversely associated in Japanese women but not in men. J Nutr.

